# Butyrate alleviates renal inflammation and fibrosis in a rat model of polycystic ovarian syndrome by suppression of SDF-1

**DOI:** 10.1186/s40360-023-00692-9

**Published:** 2023-10-03

**Authors:** Al-Amin M. Bashir, Kehinde S. Olaniyi

**Affiliations:** Cardio/Endo-metabolic and Microbiome Research Unit, Department of Physiology, College of Medicine and Health Sciences, Afe Babalola University, Ado-Ekiti, 360101 Nigeria

**Keywords:** Butyrate, Inflammation, Kidney, PCOS, SDF-1

## Abstract

**Background:**

Polycystic ovarian syndrome (PCOS) is a multifactorial condition with metabolic-related complications, such as diabetic nephropathy and chronic renal disorder, which are the leading cause of renal transplant globally. Protective effects of histone deacetylase (HDAC) inhibitors (HDACi) have been documented in metabolic-linked pathologies. Nonetheless, the current study investigated the restorative role of HDACi, butyrate in experimental PCOS-induced renal disorder.

**Materials and methods:**

Female Wistar rats (8-week-old) were divided into groups; control, butyrate-treated, letrozole and letrozole + butyrate-treated groups. To induce PCOS, 1 mg/kg of letrozole was given (oral gavage) for 21 days. After confirmation of PCOS, 200 mg/kg of butyrate (oral gavage) was administered for 6 weeks.

**Results:**

Rats with PCOS revealed disruption in glucose homeostasis (hyperinsulinemia and impaired glucose tolerance and insulin resistance) and presented with the phenotypes of PCOS (hyperandrogenism, multiple ovarian cysts and elevated LH/FSH ratio). Increased plasma and renal triglycerides and inflammatory (TNF-α/SDF-1/NF-κB) markers were observed with elevated levels of TGFβ-1, renal lipid peroxidation and redox imbalance (GGT, GSH, HIF-1α). Interestingly, animals with PCOS reported increased body weight as well as renal mass. Whereas, heightened levels of plasma urea, creatinine and creatine kinase indicating renal dysfunction, characterized by renal apoptosis (Caspase-6) and increased HDAC2 levels. Notwithstanding, administration of butyrate averted the alterations.

**Conclusion:**

The present investigation demonstrates that PCOS declines renal function, which is accompanied by renal inflammation, apoptosis and fibrosis. The study further suggests that butyrate, an HDAC2i restores renal function by suppressing renal SDF-1 with subsequent attenuation of renal inflammation, apoptosis and fibrosis.

## Introduction

Polycystic ovarian syndrome (PCOS) is a multifactorial endocrine disorder [1] recognized as one of the most common reproductive-metabolic disorder of child bearing-aged women. Its major endocrine and metabolic manifestations include hirsutism, oligo-anovulation, and polycystic ovarian morphology upon ultrasound imaging which are in association with glucose intolerance and dyslipidemia [2]. This disorder is of public health concern and affects 6–21% of women of reproductive age worldwide [3]. While a multitude of reproductive and metabolic abnormalities associate with PCOS, its convoluted etiology, presumably multifactorial and heterogeneous, is still not completely known [4].

Polycystic ovarian syndrome exhibits fundamental manifestations of metabolic syndrome including insulin resistance (IR). It is found to be the major concern of reproductive health in women and puts serious life-threatening conditions. The mechanistic link between PCOS in women of reproductive age and renal disorder remains elusive. Chronic kidney disease (CKD) is predominant in 10% of the world’s adult population, and is increasingly considered a silent epidemic [5]. Although a number of studies have unraveled the involvement of insulin resistance and inflammation and this has been implicated in contributing to podocyte injury [6]. Hyperinsulinemia caused by tissue IR is central to PCOS pathology [7]. Studies have suggested that individuals with metabolic syndrome are also at risk for developing CKD reflected by renal dysfunction. Even in the absence of other co-existing metabolic syndrome components, inflammatory mediators alone can trigger IR, as seen in uremia, possibly causing IR by disrupting insulin signaling [8].

Studies have shown that impaired renal function in CKD is usually characterized by disrupted microvascular architecture and accumulation of fibrotic matrix, which has been linked to angiogenic pathway of which the stromal cell-derived factor-1 (SDF-1) is critical [9]. Stromal cell-derived factor-1 is a CXC chemokine and the major ligand for chemokine receptor type 4 (CXCR4), a seven transmembrane domain G-protein coupled receptor that is found in endothelial cells, and also localized in the podocytes and distal tubular cells of the kidney with multiple contextual functions [10]. However, hyperproliferation of SDF-1 has been reported in the development of glomerular disease or kidney disease, especially in metabolic-related disease such as type 2 diabetes mellitus [11]. In addition, elevated level of SDF-1 induces inflammation through the activation of a non-canonical NF-κB signaling pathway in the kidney [12], promoting glomerular sclerosis, loss of podocytes and apoptosis [13]. Similarly, NF-κB is a family of inducible transcription factors responsible for regulating the induction and progression of inflammatory responses in many diseases, including CKD [14]. Recent study by Ye et al., reported that activation of NF-κB signaling promotes renal tubular cell apoptosis, inflammation and fibrosis in the kidneys of PCOS mouse model, which was significantly correlated with elevated level of tumor necrosis factor- α (TNF-α) [15]. Therefore, it appears that by attenuating SDF-1-induced NF-κB-dependent pathway may have a positive effect on the treatment of PCOS-associated renal disorder.

Epigenetic changes are pervasive in kidney diseases and likely responsible as a source of phenotypic variation [16]. Prior evidence has demonstrated the involvement of histone deacetylase (HDAC) in the progression of various metabolic disorders, suggesting that HDAC inhibitors (HDACi) might be an emerging treatment agent for reno-metabolic associated disorders [17]. A study by Andrande-Oliveira et al., implicated short chain fatty acids (SCFA) as modulators in inflammatory processes, increasing tubular proliferating cells, and enhancing autophagy [18]. Short chain fatty are the main metabolites produced in the colon by bacterial fermentation of dietary fibers and resistant starch and these include butyrate, acetate and propionate [19]. Butyrate has been well documented as HDACi [20, 21], and its anti-inflammatory property has been demonstrated in cisplatin-induced renal injuries [22]. Howbeit, the present study was designed to investigate the impact of HDACi, butyrate on renal deficit in experimental PCOS rat model. The study in addition evaluated the probable involvement of SDF-1.

## Materials and methods

### Experimental animals and grouping

The study was carried out and reported in accordance with the ARRIVE guidelines. Eight-week-old female Wistar rats with average body weight of 151.33 ± 3.30 g was procured from the animal house of College of Medicine and Health Sciences, Afe Babalola University, Nigeria. The rats were allowed to have free access to standard rat chow and tap water. The rats used for this study were with at least three consecutive regular estrous cycles with the same estrous stage, which was determined through vaginal smear. The rats were acclimatizing for a week and assigned randomly into four groups with n = 5 per group; control (CTL), butyrate (BUT)-treated, letrozole (LET) and LET + BUT groups. Rats were maintained in a colony under standard environmental conditions (22–26 ^0^ C of temperature), (50–60% of relative humidity), and 12-hour dark/light cycle.

### Induction and confirmation of PCOS

Polycystic ovarian syndrome was induced in experimental rats through uninterrupted administration of 1 mg/kg body weight of letrozole (oral gavage; Sigma-Aldrich, St Louis, MI.) once daily for a period of 21 days as previously documented [23, 24]. Manifestation of PCOS was confirmed using Rotterdam criteria [25] by monitoring the estrous cycle and testosterone level. Histology of the ovaries was performed at the end of the treatments.

### Treatment

Control group received distilled water (*p.o.*) as vehicle, 200 mg/kg of sodium butyrate (oral gavage; Sigma-Aldrich, St Louis, MI) was given to BUT group, while LET group received distilled water (oral gavage) and LET + BUT group received sodium butyrate (oral gavage) with the administration lasting for six weeks [26, 27]. Initial and final body weights were monitored with weighing balance and the difference was estimated as body weight gain.

### Assessment of glucoregulatory parameters

This was determined by performing oral glucose tolerance test (OGTT). It was performed 48 h before the sacrifice of the rats. After 12-h overnight fast, basal blood glucose was determined, and the rat were loaded by oral gavage with 2 g/kg of glucose. Then blood was obtained sequentially at 30, 60, 90 and 120 min. A hand-held glucometer was used to monitor the blood glucose levels.

### Collection of samples

At the completion of treatment, the rats were fasted overnight. Thereafter, the rats were sacrificed using anesthetic agent, 50 mg/kg body weight of sodium pentobarbital (*ip*) as a chemical method of euthanasia as previously reported [24, 26]. The blood sample was collected through cardiac puncture into heparinized tube and centrifuged at 704 g for 5 min at room temperature. Plasma was stored at -80 °C until the period of biochemical assays.

### Preparation of renal tissue homogenate

The kidneys were isolated and weighed, and 100 mg section of the tissue was carefully removed and homogenized with a glass homogenizer in phosphate buffer solution, centrifuged at 8000 g for 10 min at 4 °C and the supernatant was collected and stored at -80 °C until it is required for biochemical assays. Renal mass was determined by dividing the absolute kidney weight with body weight.

### Analysis of biochemical parameters

#### Determination of hormonal profile

Plasma concentration of insulin and testosterone were determined using rat ELISA kits purchased from Calbiotech Inc. (Cordell Ct., El Cajon, CA 92,020, USA) with cat. number IS130D and TE187S respectively. Plasma luteinizing hormone (LH) and follicle stimulating hormone (FSH) were measured using rat ELISA kit obtained from Calbiotech Inc. (Cordell Ct., El Cajon, CA 92,020, USA) with cat number LH231F and FS232F respectively. Thereafter, LH/FSH ratio was estimated.

#### Determination of triglyceride

By using the assay kits obtained from Fortress Diagnostics Ltd. (Antrium, UK) with cat number BXC0271, the levels of triglyceride (TG) were determined in the supernatants of the plasma and renal tissue.

#### Assessment of pro-inflammatory mediators, redox status and angiogenic factor

The levels of NF-κB-p65, TNF- α and SDF-1 were determined in the supernatants of the renal tissue homogenates by quantitative standard sandwich ELISA technique using rat kits obtained from Elabscience Biotechnology Inc. (Wuhan, Hubei, P.R.C., China) with cat number E-EL-R067496T, E-EL-R001996T and E-EL-R3027 respectively. Similarly, the level of malondialdehyde (MDA) was determined by standard non-enzymatic spectrophotometric method using assay kits from Randox Laboratory Ltd. (Co. Antrim, UK). The glutathione (GSH) level was determined in the renal tissue using a non-enzymatic spectrophotometric method with assay kits obtained from Oxford Biomedical Research Inc. (Oxford, USA). In addition, the levels of hypoxia inducible factor-1α (HIF-1α) and tissue growth factor β-1 (TGFβ-1) were determined using rat ELISA kits purchased from Elabscience Biotechnology Inc. (Wuhan, Hubei, P.R.C., China) with cat number E-EL-R0513 and E-AB-22,214 respectively, in compliance with the manufacturer’s procedures.

#### Determination of caspase-6 and γ-Glutamyl transferase (GGT)

The concentration of caspase-6 was determined from the renal tissue using rat ELISA kit, obtained from ELK Biotechnology Co. Ltd. (1312 17th Street #692 Denver, CO 80,202 USA) with cat number ELK8812, in compliance with the manufacturer’s procedure. In addition, GGT activities were determined from the renal tissues by standardized enzymatic colorimetric method using assay kits obtained from Randox Laboratory Ltd. (Co. Antrim, UK).

#### Assessment of renal function markers

By using reagents obtained from Randox Laboratory Ltd. (Co. Antrim, UK), plasma creatinine and urea were determined by standardized non-enzymatic colorimetric methods. Urea/creatinine ratio was estimated and the level of creatine kinase was determined using rat ELISA kits obtained from Elabscience Biotechnology Inc. (Wuhan, Hubei, P.R.C., China, cat number: E-EL-R0274).

#### Determination of histone deacetylase-2 level

The renal tissue level of HDAC2 was determined using Rat ELISA kits obtained from ELK Biotechnology Co. Ltd. (1312 17th Street #692 Denver, CO 80,202 USA) with cat number ELK7409, in compliance with the manufacturer’s procedure.

#### Histological evaluation of ovaries and kidneys

Hematoxylin and Eosin and Masson’s Trichrome staining techniques were used for histological evaluation of ovaries and kidneys respectively. A section of the ovary and kidney were fixed in 10% formalin overnight and dehydrated, embedded in paraffin, and sectioned at 5-µm thickness as previously described [28, 29]. The slides were prepared and OPTO-Edu industrial camera light microscope and a computer (Nikon, Japan) were used to examined the slides.

### Analysis of data

The normality of data was tested using Shapiro-Wilk test and the data were normally distributed. Statistical group analysis was performed with GraphPad Prism software version 9, and all data were expressed as means ± SD. One-way ANOVA or repeated ANOVA as appropriate was used to compare the mean values of variables among the groups. Bonferroni’s test was used for *post hoc* analysis. Statistically significant difference was considered at p less than 0.05.

## Results

### Effects of butyrate on body weight and renal mass in letrozole-induced PCOS

There was a significant increase (p < 0.05) in body weight and the kidney mass in animals with PCOS compared with CTL and this was normalized (p < 0.05) in PCOS animals treated with BUT when compared with untreated LET group (Table 1).

**Table 1:** Body weight and renal mass

GROUPS	CTL	BUT	LET	LET + BUT
**Initial Body weight (g)**	131.20 ± 3.90	133.80 ± 4.76	142.40 ± 3.50	131.80 ± 3.08
**Body weight gain (g)**	26.40 ± 2.59	35.67 ± 1.73	48.40 ± 5.95*	39.60 ± 6.29^#^
**Renal mass (g/kg)**	6.14 ± 0.08	6.13 ± 0.13	6.55 ± 0.10*	5.98 ± 0.17^#^

Data are expressed as mean ± S.D. *n* = 5. Data were analyzed by one-way ANOVA followed by Bonferroni *post hoc* test. (*P < 0.05 vs. CTL; ^#^*P* < 0.05 vs. LET). (Control (CTL); Butyrate (BUT); Letrozole (LET).

### Effects of butyrate on glucose regulation in letrozole-induced PCOS

**Fig. 1:**
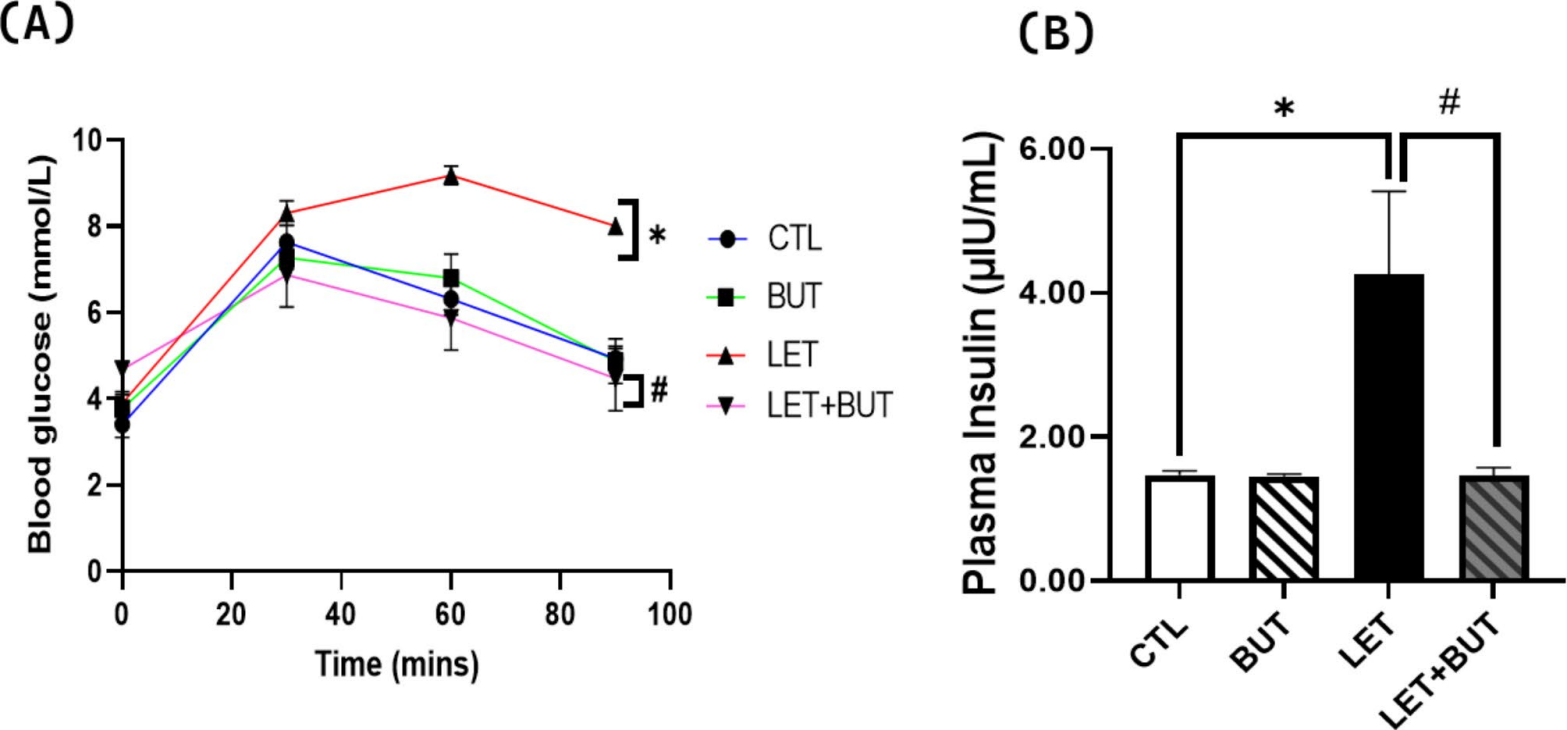
Effect of butyrate on oral glucose tolerance (**a**) and plasma insulin (**b**) in letrozole-induced PCOS Wistar rats. Data are expressed as mean ± S.D. *n* = 5. Data were analyzed by one-way ANOVA, while repeated ANOVA was used for OGTT data followed by Bonferroni *post hoc* test. (*P < 0.05 vs. CTL; ^#^*P* < 0.05 vs. LET). Control (CTL); Butyrate (BUT); Letrozole (LET); Oral glucose tolerance test OGTT).

Significantly elevated (p < 0.05) levels of plasma insulin and impaired glucose tolerance were observed in PCOS rats in addition with increased blood glucose levels when compared to control. However, consequent treatment with BUT resulted in a notable reduction (p < 0.05) in these parameters (Fig. 1).

### Effects of butyrate on endocrine parameters and ovarian morphology in letrozole-induced PCOS rats

**Fig. 2:**
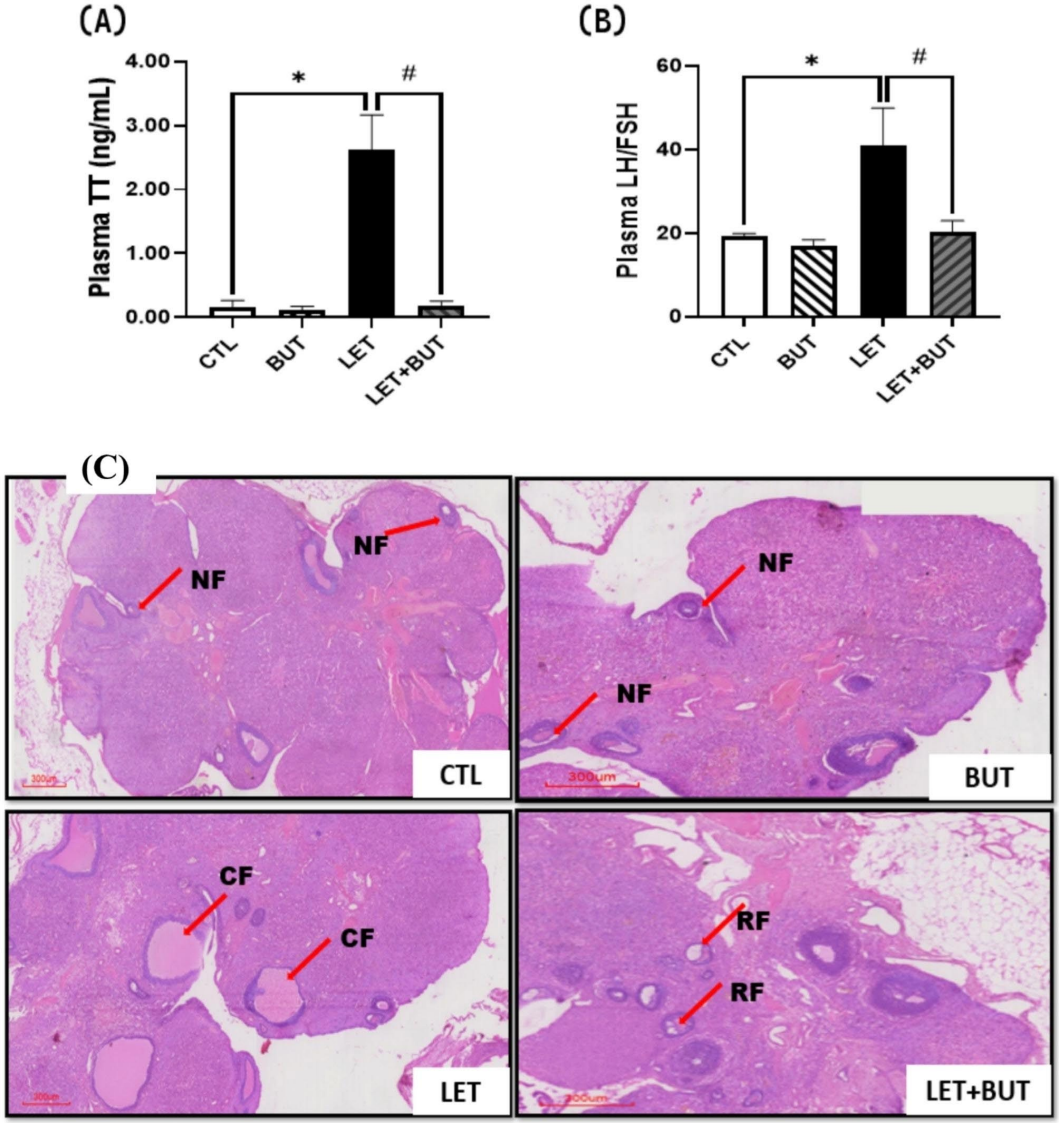
Effect of butyrate (BUT) on plasma testosterone (**a**) and LH/FSH (**b**) and ovarian morphology (**c**) in letrozole-induced PCOS rats. Data are expressed as mean ± S.D. *n* = 5. Data were analyzed by one-way ANOVA followed by Bonferroni *post hoc* test. (*P < 0.05 vs. CTL; ^#^*P* < 0.05 vs. LET). (Scale Bar: 300 μm). Control (CTL); Butyrate (BUT); Letrozole (LET); Testosterone (TT); Luteinizing hormone (LH); Follicle stimulating hormone (FSH); Normal follicle (NF); Cystic follicle (CF); Restored follicle (RF).

Circulating testosterone and the LH/FSH ratio showed a significant increase (p < 0.05) in PCOS rats. However, BUT reduced (p < 0.05) the hormonal parameters in PCOS animals. In addition, ovarian histology of PCOS rats were characterized with multiple cysts, which were attenuated following administration of BUT in LET + BUT group (Fig. 2).

### Effects of butyrate on plasma and renal triglyceride in letrozole-induced PCOS

**Fig. 3:**
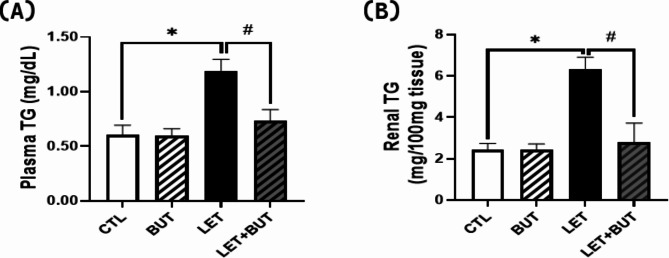
Effects of butyrate (BUT) on plasma and renal triglyceride (**a, b**) in letrozole-induced PCOS rats. Data are expressed as mean ± S.D. *n* = 5. Data were analyzed by one-way ANOVA followed by Bonferroni *post hoc* test. (*P < 0.05 vs. CTL; ^#^*P* < 0.05 vs. LET). Control (CTL); Butyrate (BUT); Letrozole (LET); Triglyceride (TG).

There was a significant increase (p < 0.05) in plasma and renal TG in animals with PCOS when compared to control, this was reduced (p < 0.05) upon administration of BUT (Fig. 3).

### Effect of butyrate on inflammatory mediators in letrozole-induced PCOS rats

**Fig. 4:**
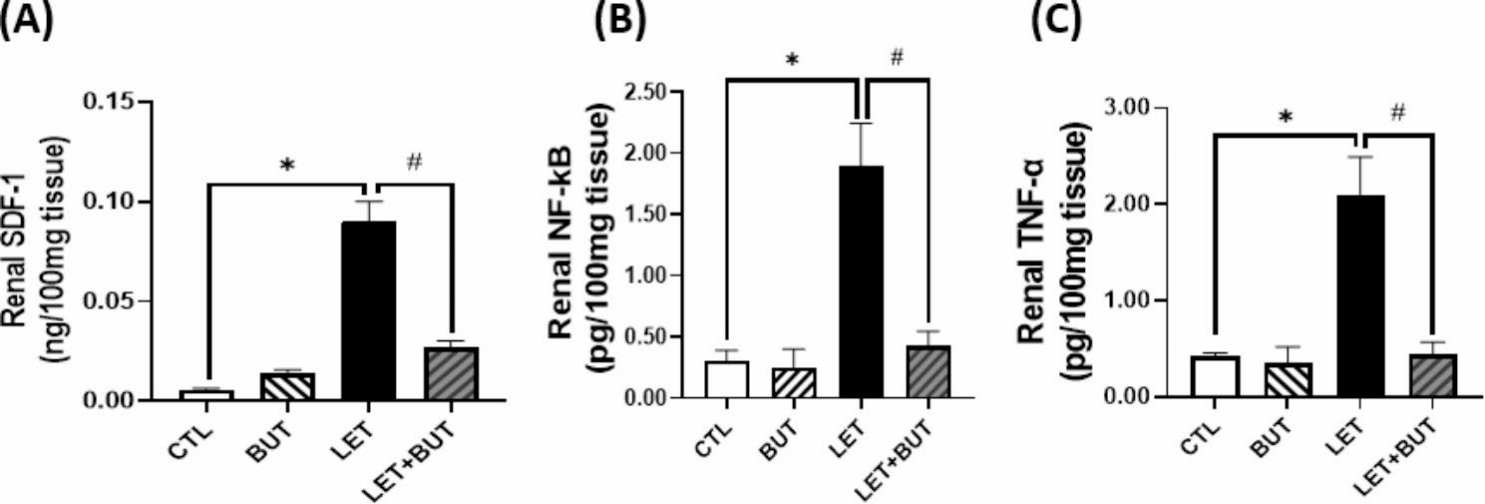
Effects of butyrate (BUT) on inflammatory parameters (**a-c**) in letrozole-induced PCOS rats. Data are expressed as mean ± S.D. *n* = 5. Data were analyzed by one-way ANOVA followed by Bonferroni *post hoc* test. (*P < 0.05 vs. CTL; ^#^*P* < 0.05 vs. LET). Control (CTL); Butyrate (BUT); Letrozole (LET); Stromal cell derived factor (SDF-1); Nuclear factor kappa B (NF-κB); Tumor necrosis factor alpha (TNF-α).

Renal SDF-1, NF-κB and TNF-α were significantly increased (p < 0.05) in LET animals. Conversely, these markers of inflammation were decreased following the administration of BUT (Fig. 4).

### Effect of butyrate on lipid peroxidation, anti-oxidant system and angiogenic factor in letrozole-induced PCOS

**Fig. 5:**
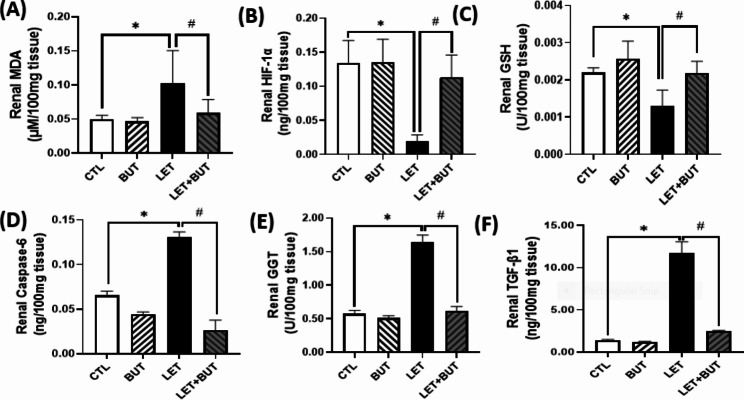
Effects of butyrate (BUT) on MDA (**a**), HIF (**b**), GSH (**c**), caspase-6 (**d**), GGT (**e**), and TGF-β1 (**f**) in letrozole-induced PCOS rats. Data are expressed as mean ± S.D. *n* = 5. Data were analyzed by one-way ANOVA followed by Bonferroni *post hoc* test. (*P < 0.05 vs. CTL; ^#^*P* < 0.05 vs. LET). Control (CTL); Butyrate (BUT); Letrozole (LET); Malondialdehyde (MDA); Hypoxia-inducible factor-1 alpha (HIF1α); Gamma-glutamyl transferase (GGT); Transforming growth factor beta-1 (TGFβ-1); Reduced glutathione (GSH).

The animal model of PCOS significantly increased (p < 0.05) renal caspase-6, GGT, TGFβ-1 and MDA when compared with control, which were decreased (p < 0.05) by BUT. Notwithstanding, renal HIF-1 and GSH were significantly reduced in PCOS rats when compared with control, alterations in these parameters improved upon BUT administration (Fig. 5).

### Effect of butyrate on renal function markers in letrozole-induced PCOS

**Fig. 6:**
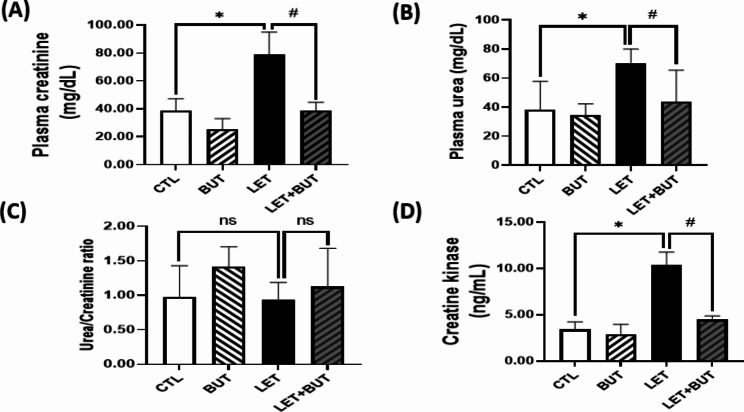
Effects of butyrate (BUT) on renal function markers in letrozole-induced PCOS rats. Data are expressed as mean ± S.D. *n* = 5. Data were analyzed by one-way ANOVA followed by Bonferroni *post hoc* test. (*P < 0.05 vs. CTL; ^#^*P* < 0.05 vs. LET). Control (CTL); Butyrate (BUT); Letrozole (LET).

There was a significant increase (p < 0.05) in plasma urea and creatinine concentration and in creatine kinase in PCOS animals when compared with control animals. But in observing the urea/creatinine ratio, no significant ratio was reported when compared with control animals. Notwithstanding, administration of butyrate significantly reduced (p < 0.05) urea concentration and creatinine concentration and creatine kinase in LET + BUT animals when compared with untreated PCOS animals (Fig. 6).

### Effect of butyrate on histone deacetylase-2 in letrozole-induced PCOS

**Fig. 7:**
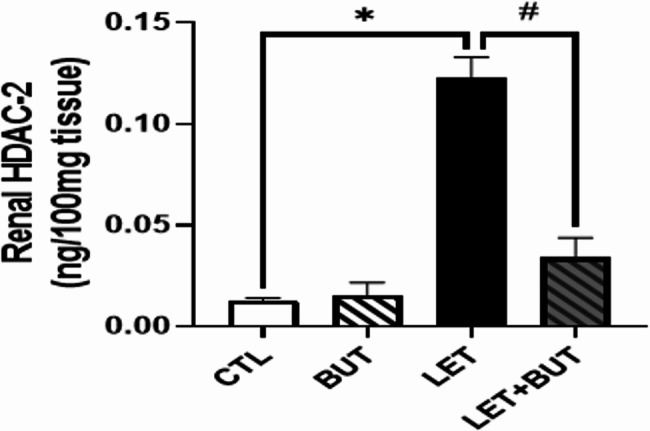
Effect of histone deacetylase inhibition, butyrate (BUT) on HDAC2 in letrozole-induced PCOS rats. Data are expressed as mean ± S.D. *n* = 5. Data were analyzed by one-way ANOVA followed by Bonferroni *post hoc* test. (*P < 0.05 vs. CTL; ^#^*P* < 0.05 vs. LET). Histone deacetylase-2 (HDAC2); Control (CTL); Butyrate (BUT); Letrozole (LET).

In PCOS animals, HDAC2 levels was found to be significantly increased (p < 0.05). Consequently, this was notably reduced (p < 0.05) following BUT administration (Fig. 7).

### Effect of butyrate on renal morphology in letrozole-induced PCOS

**Fig. 8:**
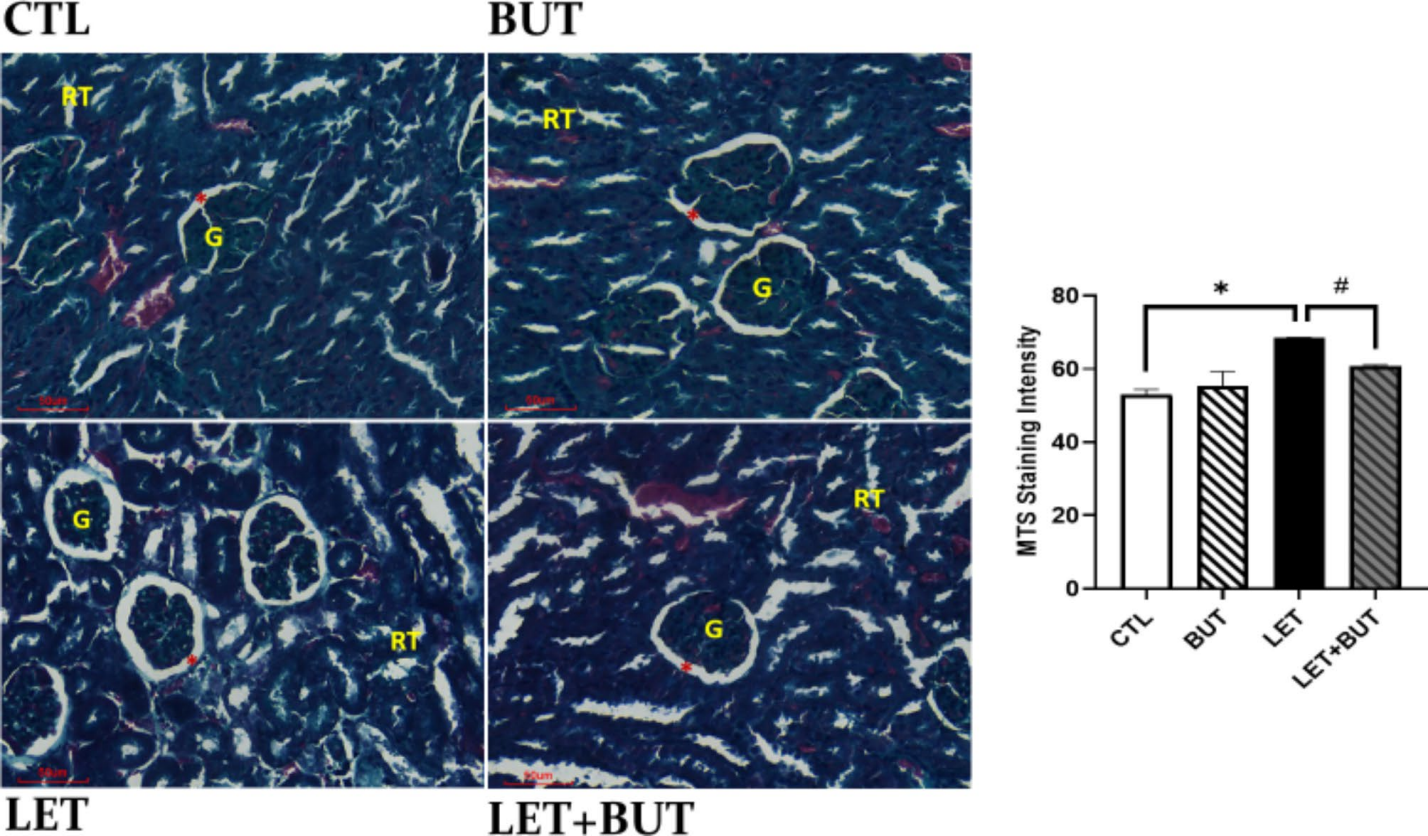
Photomicrograph showing the histology of the kidney stained with Masson’s trichrome. (Scale Bar: 50 μm). (*P < 0.05 vs. CTL; ^#^*P* < 0.05 vs. LET). (G: glomerulus, RT: Renal tubules; Red Star: Urinary Space). Control (CTL); Butyrate (BUT); Letrozole (LET).

Photomicrograph showing the histology of the kidney stained with Masson’s trichrome. Control group revealed normal degree/quantum of collagen in the glomeruli and renal tubules region. Butyrate group showed normal collagen deposition while letrozole group revealed increased collagen deposit in the intra-glomerular region, enlargement of the bowman’s space as a result of decrease in glomeruli. LET + BUT revealed statistically significant decrease in collagen deposition when compared with LET group (p < 0.05) (Fig. 8).

## Discussion

The primary finding of the current study demonstrates that HDAC2i, butyrate alleviates renal fibrosis and/or inflammation in PCOS rat model. In addition, the results reported an increased renal mass and body weight gain, as well as disrupted glucose regulation (hyperinsulinemia and impaired glucose tolerance) in animals with PCOS phenotypes (hyperandrogenism, multiple ovarian cysts and elevated LH/FSH ratio). In addition, the PCOS animals also showed elevated renal TG and redox imbalance (MDA, GGT, GSH, HIF-1α) which subsequently led to renal apoptosis (caspase-6) and thus precipitating renal dysfunction (urea, creatinine and creatine kinase) in PCOS animals. However, administration of butyrate, an HDAC2 inhibitor reversed the renal disturbance in PCOS rat model.

The pursuit in finding the precise pathophysiological mechanism on which PCOS operates remains ongoing. Our data demonstrated body weight gain, increased renal mass and reduced insulin sensitivity with impaired glucose tolerance in PCOS animals, which validated the metabolic features of PCOS, as earlier reported that women with PCOS manifest metabolic abnormalities [28, 29]. Likewise, an increase in renal mass as observed in PCOS animals is a pointer to kidney hypertrophy and this occurs in many renal disorders, especially metabolic-driven disorder [30]. The increase in renal mass is predominantly due to proximal tubular epithelial cell hypertrophy [31]. Moreover, the present study reveals renal hypertrophy, which is an early characteristic pathological change that occurs in diabetic nephropathy and is closely associated with late renal fibrosis [32]. However, supplementation of butyrate was found to have metabolic benefits, which is evident with the significant reduction of glucose dysregulation, body weight and renal mass. Impaired glucose tolerance resulting from insulin resistance is a characteristic manifestation of metabolic-driven kidney disease [33]. This study showed disruption in glucose regulation as revealed by impaired oral glucose tolerance in PCOS animals compared with control and this is consistent with earlier observation [34]. This contributes to renal lipotoxicity, characterized with excessive lipid accumulation (renal TG), which was attenuated when supplemented with butyrate as shown in LET + BUT group compared with untreated LET group. Furthermore, higher androgen levels in PCOS may cause podocyte damage thus directly impacting development of kidney damage [35], and the level of androgen (testosterone) was significantly decreased in LET group following treatment with butyrate compared with untreated LET group. Decreased level of androgen by butyrate also led to an improvement in glucose regulation with subsequent increase in insulin sensitivity, thus attenuating excess body weight and renal lipid deposition with consequent decrease in renal mass. These observations validated the anti-androgenic and metabolic benefits of SCFAs, including butyrate in metabolic-related pathologies [17, 36].

In addition, excessive lipid deposition in the renal tissue of PCOS animals possibly contributes to renal hypertrophy observed in the present study. This renal event often triggers cellular response that decreases antioxidant defense due to increased lipid peroxidation as observed in PCOS animals with an imbalance between oxidant (MDA) and antioxidant (GSH), causing oxidative stress in the renal tissue. Oxidative stress exposes the cellular structures to injury, thereby inducing SDF-1-dependent signaling pathway to promote angiogenesis [9, 37]. Similar to earlier observation, SDF-1 significantly increased in the kidney of PCOS animals compared with control. Increased level of SDF-1 has also been documented to cause inflammation through the activation of NF-κB-dependent mechanism [12], which is validated in the present study with a significant increase in renal NF-κB level. These observations suggest that PCOS-induced elevated SDF-1 is accompanied by renal inflammation, which is mediated by increased NF-κB and TNF-α. The renal inflammation further deteriorates to cellular apoptosis as revealed by increased level of caspase-6 in PCOS animals, thus contributing to renal injury (elevated GGT) and declined renal function with corresponding increase in plasma creatinine, urea and creatine kinase, which are potent markers of renal function. Although urea/creatinine ratio was not significantly altered in PCOS animals when compared with control, which indicates that PCOS-induced renal injury/damage is not associated with prerenal cause, but possibly linked to the alteration of intrarenal cellular and biochemical components or intrarenal pathology. As previously reported, elevated urea/creatinine ratio is an indicator of prerenal pathology [38]. However, administration of butyrate to PCOS animals as shown in LET + BUT group significantly reduced renal oxidative stress by improving antioxidant defense (GSH) and decreased inflammation by attenuating hyperproliferated SDF-1 with corresponding decrease in NF-κB and TNF-α. These subsequently mitigate cellular apoptosis/injury and restore renal function as shown with significant decrease in caspase-6/GGT and renal function markers respectively in LET + BUT group compared with untreated LET group. Over all, these demonstrate the antioxidant, anti-inflammatory and anti-apoptotic properties of butyrate against renal damage in PCOS model. The above effects of butyrate are similar to earlier studies that documented the antioxidant and anti-inflammatory impacts of SCFAs, including butyrate in [18, 19, 22] in non-metabolic-driven pathologies.

Additionally, SDF-1 being an angiogenic factor could compensatorily respond to inflammation-induced suppressed level of HIF-1α in the kidney of PCOS animals, thereby promoting hyperproliferation of SDF-1 with corresponding increase in TGF-β. Thus, contributing to the accumulation of fibrotic matrix in the renal tissue of PCOS animals, which was histologically confirmed using Masson trichrome staining technique, and this subsequently resulted in renal deficit. Hence, the present observations are consistent with previous studies, which reported that increased renal tissue fibrosis contributes to renal dysfunction in CKD [9]. Similarly, earlier study in PCOS mouse model has also documented the development of tubular cellular apoptosis and fibrosis in the kidney [15], which is also in consonance with our present observation. Therefore, our present findings collectively suggest that PCOS causes renal fibrosis with a consequent decline in renal function, a detrimental effect that is possibly mediated by SDF-1-induced inflammation. Nevertheless, administration of butyrate blotted renal fibrosis and restored renal function by suppressing SDF-1-induced inflammation and TGF-β as well as improving HIF-1α. Our observations are also similar to the previous studies, which reported that HDAC inhibitor downregulate angiogenesis [39]. HDACi can hyperacetylate hypoxia-inducible factor (HIF-1α) [39], thereby improving a pro-angiogenic transcription factor, such as SDF-1 as demonstrated in the present study, thus suppressing inflammation, fibrosis and cellular apoptosis in the renal tissue of PCOS animals. Moreover, the present result also showed a significant decrease in the level of HDAC2 in the renal tissue of PCOS animals with corresponding decrease in SDF-1, thereby attenuating inflammation, fibrosis and apoptosis after treatment with butyrate compared with untreated PCOS (LET) group. These observations seem similar to the previous study by Advani et al., who reported that HDAC inhibition attenuated glomerular fibrotic matrix accumulation in diabetic mice [40]. Hence, the above findings suggest that butyrate, an HDAC2i ameliorates renal inflammation and fibrosis with restoration of renal function in PCOS rat model, which is accompanied by suppression of SDF-1.

## Conclusion

The present investigation demonstrates that PCOS declines renal function, which is accompanied by renal inflammation, apoptosis and fibrosis. The study further suggests that butyrate, an HDAC2i restores renal function by suppressing renal SDF-1 with subsequent attenuation of renal inflammation, apoptosis and fibrosis.

## Data Availability

The data supporting the present study will be made available from the corresponding author on request.

## Abbreviations

BUT: Butyrate
(Cat): Catalog
CKD: Chronic kidney disease
FSH: Follicle stimulating hormone
GTT: Gamma-glutamyl transferase
GSH: Reduced glutathione
HDAC: Histone deacetylase
HIF: Hypoxia-inducible factor-1
IR: Insulin resistance
LET: Letrozole
LH: Luteinizing hormone
MDA: Malondialdehyde
NF-κ B: Nuclear factor-kappaB
PCOS: Polycystic ovarian syndrome
SDF-1: Stromal cell derived factor-1
TGF-β1: Transforming growth factor- β1
TNF-α: Tumor necrosis factor-α
TG: Triglyceride
